# Pathway and Mechanism for the Biodegradation of Inosine and Guanosine by *Heyndrickxia coagulans* NJ01

**DOI:** 10.3390/foods15183254

**Published:** 2026-09-15

**Authors:** Zhenyu Yuan, Xiaoyu Cao, Qianqian Xu, Kun Lin, Xinyue Du, Hai Yan

**Affiliations:** School of Chemistry and Biological Engineering, University of Science and Technology Beijing, Beijing 100083, China; m202421167@xs.ustb.edu.cn (Z.Y.); xiaoyucao98@ustb.edu.cn (X.C.);

**Keywords:** Hyperuricemia, *Heyndrickxia coagulans* NJ01, biodegradation, whole-genome sequencing, metabolic pathway

## Abstract

Hyperuricemia (HUA) and gout are primarily caused by excessive uric acid production resulting from the catabolism of purine nucleosides, notably inosine and guanosine. In this study, a candidate probiotic strain capable of biodegrading nucleosides (both inosine and guanosine) was successfully isolated from *Jiaosu* and identified as *Heyndrickxia coagulans* NJ01 by average nucleotide identity analysis. In vitro assays showed 100% biodegradation of 1.0 g/L of both inosine and guanosine by NJ01 cells and its cell-free extracts within 24 h. HPLC and genomic analyses confirmed that inosine and guanosine were biodegraded by purine-nucleoside phosphorylase into hypoxanthine and guanine, which are terminal metabolites not further degradable by the strain, and the key gene was confirmed by PCR. Furthermore, evaluation of the probiotic properties and safety of NJ01 at both genotypic and phenotypic levels confirmed that this strain is a promising probiotic candidate for further in vivo validation. This study provides a crucial foundation for the potential application of *H. coagulans* NJ01, a strain capable of biodegrading uric acid precursors, in the development of functional foods for HUA management.

## 1. Introduction

Hyperuricemia (HUA), characterized by elevated serum uric acid levels, has emerged as a significant metabolic and public health challenge worldwide [1]. HUA is a well-established cause of gout and an independent risk factor for chronic kidney disease, hypertension, and cardiovascular disorders [2]. Current clinical management primarily relies on long-term pharmacotherapy, which may be associated with adverse effects and compliance issues [3]. Dietary purine restriction, another cornerstone of management, is often difficult to maintain and may compromise nutritional balance [4]. It is therefore imperative to develop safe, effective, and sustainable dietary strategies for HUA management. In this context, the gut microbiota has emerged as a crucial player in host purine metabolism [5]. Evidence indicates probiotics may lower uric acid through multiple pathways: biodegrading dietary purines, inhibiting xanthine oxidase (XOD), producing uricase, and enhancing renal/excretory clearance via transporters such as ATP-binding cassette subfamily G member 2 (ABCG2) [6]. Probiotics also help reduce inflammation and oxidative stress, restore gut microbiota homeostasis, and strengthen intestinal barrier function [5]. However, research is still early, with limited strains and mechanistic understanding available.

Certain probiotic microorganisms, particularly lactic acid bacteria (LAB), can degrade exogenous purine nucleosides like inosine and guanosine in the gastrointestinal tract [7]. This metabolic trait reduces the bioavailability of precursors for endogenous uric acid synthesis, presenting a promising non-pharmacological avenue for urate control [8]. Consequently, the exploration of diverse and resilient probiotic species with robust nucleoside-biodegrading capabilities remains an active research frontier. Several probiotic strains capable of purine-nucleoside biodegradation have been documented, including *Pediococcus acidilactici* GR-5 [9] and several *Heyndrickxia coagulans* such as BC99 and TBC169. While GR-5 exhibits nucleoside-degrading capacity, it is a non-spore-forming lactic-acid bacterium with limited stress resistance. Although *H. coagulans* BC99 [10] and TBC169 [11] can mitigate HUA in animal models, their direct in vitro nucleoside-biodegradation mechanism and catalytic metabolites remain incomplete. Therefore, there remains a knowledge gap to obtain a spore-forming *H. coagulans* strain with systematically validated nucleoside-biodegrading phenotype, confirmed metabolic products, whole-genome information and in vitro safety and probiotic profiling.

Fermented foods are one of the key sources for probiotic screening, several nucleoside-degrading LAB strains from various fermented foods have been isolated [12], including *Pediococcus acidilactici* GR-5 [9], *Limosilactobacillus fermentum* JL-3 [13], *Lactobacillus brevis* DM9218 [14]. *Jiaosu*, a traditional fermented food, produced by microbial fermentation of Chinese medicinal materials or fruits, enzymatically hydrolyzes substrates to release bioactive components (polysaccharides, flavonoids, etc.) with the effects of boosting immunity, relieving fatigue, and regulating metabolism. Additionally, *P. acidilactici* GQ01 [7] isolated from natural fermented wolfberry (*Jiaosu*) also have shown the potential of improving HUA.

In this study, we isolated a novel strain, *Heyndrickxia coagulans* NJ01, from traditional fermented *Jiaosu*, and hypothesized that it possesses both potent nucleoside-biodegrading activity and favorable probiotic characteristics. To test this, we performed genomic sequencing to identify genes involved in purine nucleoside metabolism, and systematically evaluated its probiotic properties and safety in vitro, including aggregation and cell-surface hydrophobicity, Hemolytic activity, biochemical identification, cytotoxicity and antimicrobial activity. This work lays a solid foundation for the future development of NJ01 as a functional food or dietary supplement candidate for managing HUA.

## 2. Materials and Methods

### 2.1. Materials

*Jiaosu* was obtained from a local market (Yantai, Shandong province, China). Inosine and guanosine, each with the purity above 99%, were obtained from Shanghai Aladdin Biochemical Technology Co., Ltd. (Shanghai, China). Yeast Peptone Dextrose Medium (YPD) and Minimal Salt Medium (MSM) were procured from Solarbio Science & Technology (Beijing, China). All other chemicals were analytical or chromatographic reagents.

### 2.2. Isolation of Nucleoside-Biodegrading Strain H. coagulans NJ01

From *Jiaosu*, the target strain was isolated using a selective medium (0.51 g of MSM, 0.25 g of inosine, 0.25 g of guanosine, and 0.10 mL of trace element solution per 100 mL, with the initial pH adjusted to 7.0), in which inosine and guanosine served as the sole carbon source. The media were incubated for 3 days at 37 °C with shaking at 200 rpm. Every 3 days, 1% (*v*/*v*) cultures were subcultured into fresh selective medium under the same conditions. After 5 weeks, the final cultures were spread onto selective plates (liquid medium supplemented with 2% *w/v* agar) and then incubated at 37 °C for 48 h. After the monoclonal colony was activated in YPD medium it was then stored in 50% glycerol at −20 °C.

### 2.3. Morphological and Physiological Characterization of H. coagulans NJ01

The *H. coagulans* NJ01 was diluted and spread on YPD agar plates to observe the colony characteristics. The cultured bacteria were centrifuged at 8000× *g* for 10 min, then fixed with 2.5% glutaraldehyde at 4 °C for 4 h. The samples were observed and photographed under biotype Scanning Electron Microscope (SEM, Hitachi SU8010, Hitachinaka City, Japan) [15].

The *H. coagulans* NJ01 was inoculated at 2% concentration into 20 mL of fresh YPD liquid medium and cultured at 37 °C and 200 rpm. OD_600_ and pH of the culture broth were monitored at regular time intervals. Growth and pH profiles were then generated with incubation time on the *x*-axis and OD_600_/pH values on the *y*-axis.

### 2.4. Determination of Biodegradation Capacity of Inosine and Guanosine by H. coagulans NJ01

The biodegradation capacity of inosine and guanosine of *H. coagulans* NJ01 was evaluated following the previous method with minor adjustments [16]. The *H. coagulans* NJ01 was cultured in YPD medium at 37 °C and 200 rpm for 16 h (10^8^ CFU/mL). After centrifugation (8000× *g*, 4 °C, 10 min), the cells were washed twice with phosphate-buffered saline (PBS), then suspended in 10 mL of PBS with inosine (1.0 g/L) or guanosine (1.0 g/L), incubating at 37 °C and 200 rpm for 24 h. After the same pretreatment as described for the intact cell experiments, the cell pellet was resuspended in PBS and subjected to ultrasonic lysis using an ultrasonic homogenizer (JY92-IIDN, SCIENTZ, Ningbo, China). For *H. coagulans* NJ01, the output power was set at 360 W for 25 min (10 s on, 5 s off, 5 times per 5 min cycle). All lysates were subsequently centrifuged at 8000× *g* for 10 min at 4 °C, and the resulting supernatants were harvested as cell-free extracts (CEs). Protein concentration was determined using a BCA protein assay kit (Beyotime, Shanghai, China) (protein concentration 1.80 g/L). The obtained CEs were then used for nucleoside biodegradation following the same procedure as described for the intact cell experiments. The reaction liquid was taken at different times, then diluted with 0.5 M NaOH and passed through a water-phase microporous membrane (0.22 μm). Quantitative measurement of either inosine or guanosine was performed with high performance liquid chromatography (HPLC, Shimadzu LC-20AT, Tokyo, Japan) [17].

The separation conditions for HPLC were as follows: the chromatographic column was Kromasil C18 (250 mm × 4.6 mm, 5 µm). The mobile phase was composed of 0.5% acetic acid aqueous solution and methanol (90%: 10%). The column was eluted with binary high pressure at a constant flow rate of 1.0 mL/min. The detection wavelength was 254 nm, and the column temperature was maintained at 40 °C [18]. The corresponding standard curves were linear over the tested ranges, with the following regression equations: for inosine, Y = 41,552X + 101,609 (R^2^ = 0.9965); and for guanosine, Y = 62,650X + 330,222 (R^2^ = 0.9985), where Y is the peak area and X is the substrate concentration (mg/L).

### 2.5. Whole-Genome Sequencing of H. coagulans NJ01

NucleoBond^®^ HMW DNA kit (MN NucleoBond, Düren, Germany, 740160.20) was used for high-quality genome extraction of samples. DNA concentration and purity were determined via Qubit4.0 (Thermo, Q33226, Waltham, MA, USA) and Nanodrop (SMA4000, Taiwan, China). DNA integrity was assessed by 0.75% agarose gel electrophoresis.

Whole-genome sequencing was performed using a hybrid strategy combining second-generation short-read and third-generation long-read sequencing on the Illumina NovaSeq6000 and PacBio platforms at Sangon Biotech (Shanghai, China). Raw data were subjected to quality control using Fastp (v 0.23.0) to generate clean data and quality of the data was assessed using FastQC (v 0.11.2). Initial assembly of long-read data was conducted with Canu (v 1.3) to build genomic scaffolds, followed by the incorporation of Illumina short-read data. GapFiller (v 1.11) was used to fill gaps between contigs. Sequence correction was performed using PrInSeS-G (v 1.0.0) to correct base errors and small fragment insertions/deletions during assembly, with subsequent optimization through multiple rounds of Pilon (v1.18). Following genome assembly, the quality of the genome was assessed using BUSCO (Benchmarking Universal Single-Copy Orthologs, v 4.1.4) to evaluate the completeness of conserved core genes, and CheckM (v 1.0.12) was employed to estimate both genome completeness and contamination levels based on lineage-specific marker genes. The Whole-Genome Shotgun (WGS) project has been deposited at GenBank under accession number JBSPTY000000000.

### 2.6. Genome Annotation of H. coagulans NJ01

Gene prediction was performed using NCBI PGAP to predict coding sequences (CDSs), tRNA, and rRNA. Functional annotation of the predicted protein-coding genes was performed primarily using the Clusters of Orthologous Groups of proteins (COG), Gene Ontology (GO), Kyoto Encyclopedia of Genes and Genomes (KEGG), and Carbohydrate-Active Enzymes (CAZymes) databases. Virulence Factors of Pathogenic Bacteria (VFDB) and Comprehensive Antibiotic Resistance Database (CARD) were queried to predict the virulence genes and antibiotic resistance genes.

### 2.7. Identification of H. coagulans NJ01 by Average Nucleotide Identity (ANI)

Phylogenetic analysis was conducted based on ANI compared against genomic sequences [19]. For the ANI analysis, the chromosomal sequences of eight strains were downloaded from the GenBank database. The ANI values were calculated using OrthoANI tool (https://www.ezbiocloud.net/tools/orthoani, accessed on 23 June 2026), and then a heatmap was generated to visually represent the genetic similarities among the strains based on the results. Reference strains used for ANI analysis are summarized in Table S2.

### 2.8. PCR Amplification of Functional Genes

The genomic DNA of *H. coagulans* NJ01 was used as a template to amplify gene *deoD* encoding key biodegradation enzymes. Primers used are available in Table S1. The DNA extracted from *H. coagulans* NJ01 was used for PCR amplification. The 50 µL PCR mixture contained 25 µL of 2× FidCycle (Sangon, China), 2 µL of each primer (10 µM), 20 ng of DNA template, and sterile distilled water to volume. Amplification was carried out in a thermal cycler (TIANGEN, OSE-GP-06, Beijing, China) under the following conditions: initial denaturation at 95 °C for 3 min; 35 cycles of 95 °C for 15 s, 56 °C for 15 s, and 72 °C for 30 s; and a final extension at 72 °C for 5 min [20]. The PCR products were separated by 1% agarose gel electrophoresis (BioDee, Beijing, China), stained with Dured DNA Gel Stain (Beyotime, China) and visualized under UV light.

### 2.9. Probiotic Properties of H. coagulans NJ01

#### 2.9.1. Auto/Co-Aggregation

The assays for auto/co-aggregation were performed according to the method described by Seddik et al. [21]. The *H. coagulans* NJ01 (16 h-old) was centrifuged at 8000× *g* and for 10 min. After two washes with sterile saline, it was resuspended in 10 mL sterile saline. The cultures of *Staphylococcus aureus* and *Escherichia coli* were centrifuged under the same conditions, washed twice with sterile saline, and resuspended in sterile saline. *S. aureus* and *E. coli* were added into two centrifuge tubes respectively; then the *H. coagulans* NJ01 (*v*/*v* = 1:1) were added. The incubation was performed at 37 °C, and the OD_600_ (A_t_) of the supernatant was measured at 0, 2, and 4 h. The initial OD_600_ was defined as A_0_, with the auto/co-aggregation percentage calculated as:

(1) $$\mathrm{Auto}/\text{co-aggregation}(\%)\;=\;(1\;-\;\frac{A_{t}}{A_{0}})\times 100$$

#### 2.9.2. Cell Surface Hydrophobicity

The cell surface hydrophobicity of *H. coagulans* NJ01 was determined according to the previous research method [22]. *H. coagulans* NJ01 (16 h-old) was centrifuged and washed twice with sterile saline, then resuspended in sterile saline. The bacterial suspension was mixed with ethyl acetate (*v*/*v* = 3:1) ratio thoroughly and allowed to stand at room temperature for 30 min until layered. The lower aqueous phase was collected for OD_600_ (A_t_) at 0, 2, and 4 h. The initial OD_600_ value is A_0_.

(2) $$\mathrm{Hydrophobicity}(\%)=(1-\frac{A_{t}}{A_{0}})\times 100$$

### 2.10. Safety Assessment

#### 2.10.1. Hemolysis Assay

The Columbia blood agar plate with *H. coagulans* NJ01 (16 h-old) was incubated at 37 °C for 48 h, and then the colonies were observed for signs of complete β-type hemolysis, partial α-type hemolysis, or non-hemolytic γ-type hemolysis [23].

#### 2.10.2. Antibiotic Sensitivity Assay

The *H. coagulans* NJ01 (16 h-old) was diluted to a final concentration of 10^8^ CFU/mL and uniformly spread onto YPD agar plates. A total of 9 antimicrobial susceptibility disks were selected, including tetracycline (30 μg), ciprofloxacin (5 μg), streptomycin (10 μg), clindamycin (2 μg), gentamicin (10 μg), ampicillin (10 μg), chloramphenicol (30 μg), kanamycin (30 μg), and roxithromycin (15 μg). The disks were aseptically placed on the inoculated agar plates, which were then incubated in a 37 °C incubator for 24 h. After incubation, the diameters of inhibition zones were observed and measured to assess antibiotic sensitivity according to Clinical and Laboratory Standards Institute (CLSI 2012) [24,25].

#### 2.10.3. Amino Acid Decarboxylase Assay

For the biochemical identification assay, amino acid decarboxylase activity was detected using biochemical identification tubes (Hopebio, Qingdao, China). The monoclonal colony of *H. coagulans* NJ01 was selected from the YPD agar plate using a sterilized inoculating needle and inoculated into the biochemical identification tube. Paraffin oil was added to seal the liquid surface, and the tube mouth was sealed before incubation at 37 °C for 24 h to observe color changes [26].

#### 2.10.4. Cytotoxicity Assay

The cytotoxicity of *H. coagulans* NJ01 against Caco-2 cells was conducted following a previous research method [27]. Caco-2 cells were cultivated in Minimum Essential Medium (MEM) supplemented with 20% (*v*/*v*) fetal bovine serum, and incubated at 37 °C under a 5% CO_2_ atmosphere until reaching the logarithmic growth phase. After detachment with 0.25% trypsin, the cell suspension was seeded into a 96-well microplate at a final density of approximately 20,000 cells per well, and MEM medium was added to ensure uniform distribution of cells at the bottom of the wells, followed by 24 h incubation. Meanwhile, *H. coagulans* NJ01 (16 h-old) was centrifuged at 4 °C and 8000× *g* for 10 min. 200 μL of the supernatant was added to each well containing adherent cells. Meanwhile, blank controls (MEM medium only, no cells) and cell controls (cells and MEM medium, no supernatant) were set up in parallel. After adding 10 μL of CCK-8 reagent to every 100 μL of medium in each well, the microplate was incubated for an additional 4 h under the same conditions. The values of absorbance at 450 nm were measured. Cell viability (%) was calculated as:

(3) $$\mathrm{Cell}\;\mathrm{viability}(\%)\;=\;\frac{A_{1}-A_{3}}{A_{2}-A_{3}}\times 100$$

Here, A_1_ is absorbance of treated wells; A_2_ is absorbance of cell controls; and A_3_ is absorbance of blank controls.

### 2.11. Statistical Analysis

All experiments were conducted in triplicate, and data are expressed as mean ± standard deviation (SD). Statistical analysis was performed using OriginPro 2025 and Graph Prism 9.5 software.

## 3. Results

### 3.1. Isolation, Morphological and Physiological Characterization of H. coagulans NJ01

**Figure 1:**
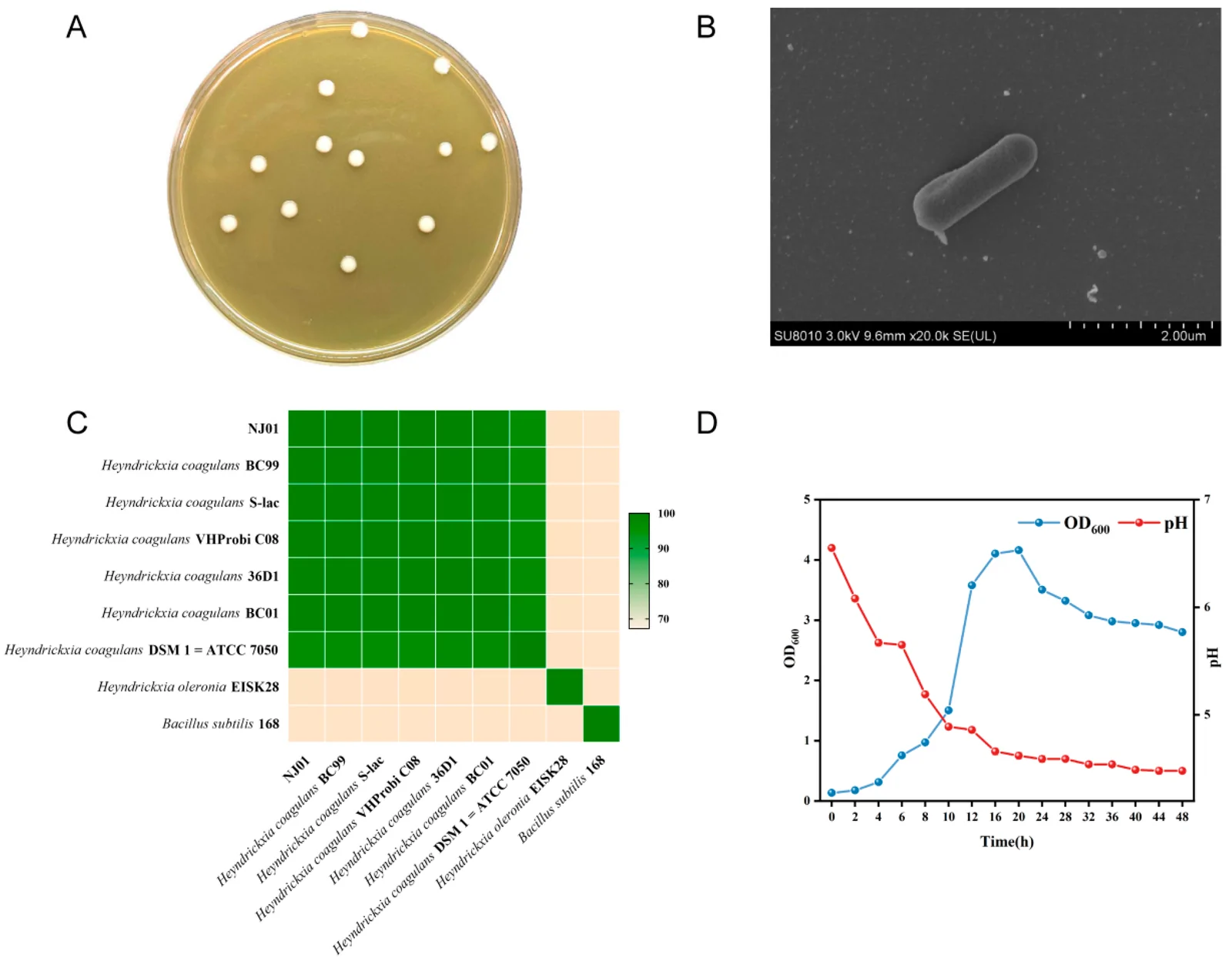
Morphological and physiological characterization of *H. coagulans* NJ01. (**A**) Single colonies were grown on a YPD agar plate. (**B**) Scanning electron microscope image of *H. coagulans* NJ01. (**C**) Average nucleotide identity analysis of *H. coagulans* NJ01. (**D**) Growth curve and acid production curve of *H. coagulans* NJ01.

The single colony of *H. coagulans* NJ01 isolated from *Jiaosu* formed milky-white, circular colonies with distinct and regular edges, and a smooth and convex surface (Figure 1A). Cells of *H. coagulans* NJ01 exhibited a rod-shaped morphology with rounded ends under SEM. The cells were typically 1–3 μm in length and approximately 0.5–1 μm in width, arranged in an orderly manner, usually existing as single cells or short chains (Figure 1B). ANI analysis revealed that the genome of NJ01 shared 99.97% homology with that of *H. coagulans* type strains, further validating its taxonomic classification as a member of *H. coagulans* (Figure 1C). OD at 600 nm and pH value were used as indicators of the growth rate and acid production capacity of *H. coagulans* NJ01, respectively. A single colony of *H. coagulans* NJ01 was inoculated into liquid YPD medium and cultured for 48 h. During this period, the maximum OD_600_ value reached 4.16, accompanied by a corresponding decrease in pH (Figure 1D). The strain of *H. coagulans* NJ01 was deposited in the China General Microbiological Culture Collection Center (CGMCC No. 37022).

### 3.2. Biodegradation of Inosine and Guanosine by H. coagulans NJ01

**Figure 2:**
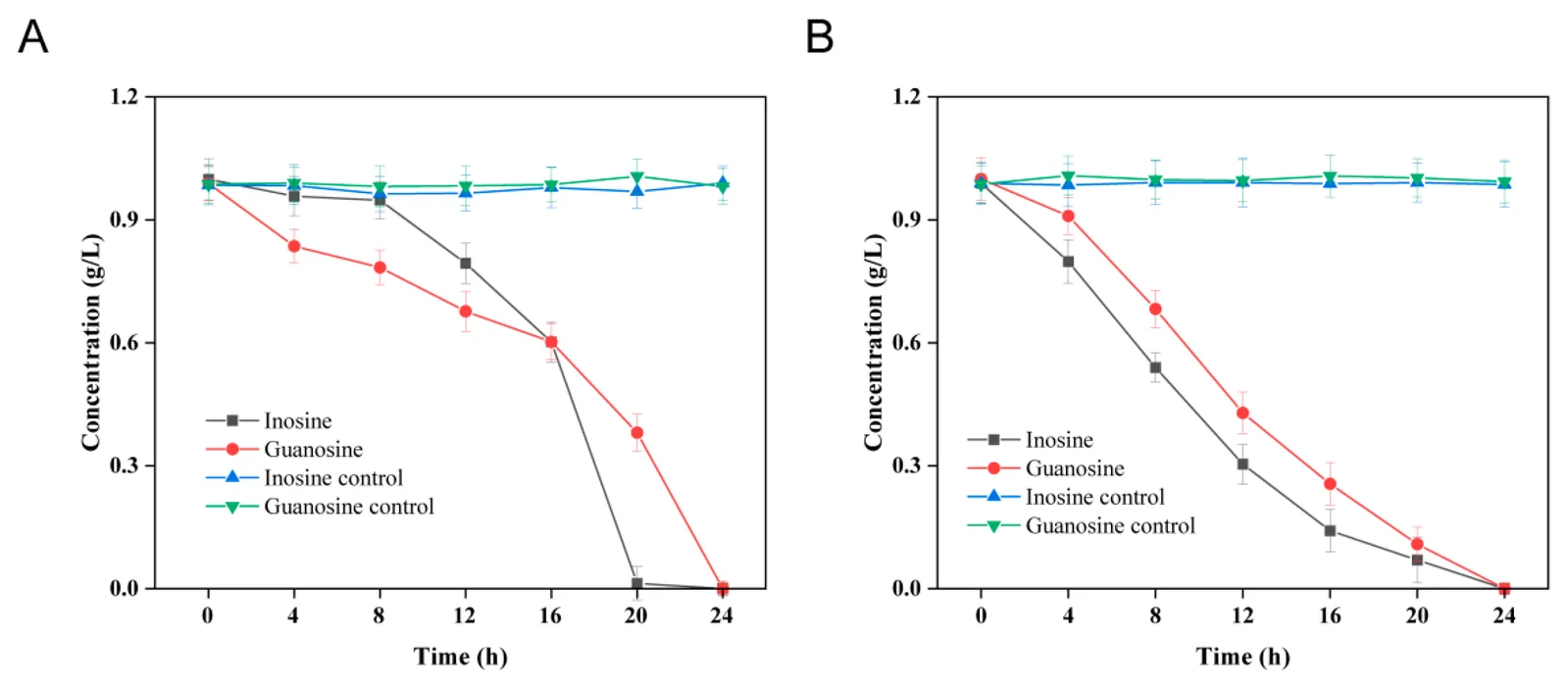
Biodegradation of inosine and guanosine by (**A**) *H*. *coagulans* NJ01 and (**B**) its CEs. Data are expressed as mean ± SD from three independent biological replicates (*n* = 3). Noninoculated medium and medium without cell-free extracts (CEs) served as controls.

To evaluate the inosine and guanosine biodegradation capacity of *H. coagulans* NJ01, this strain was inoculated into PBS supplemented with 1.0 g/L inosine or 1.0 g/L guanosine, respectively. The results demonstrated that *H. coagulans* NJ01 resting cells degraded 100% of both nucleosides within 24 h, with a significantly higher degradation rate observed for inosine compared to guanosine (Figure 2A). Furthermore, its CEs also biodegraded 100% of both nucleosides within 24 h (Figure 2B).

HPLC analysis further confirmed the biodegradation of inosine and guanosine. Specifically, HPLC chromatogram revealed the dynamic changes in the peak intensities of inosine, guanosine, and their respective metabolic products throughout the entire biodegradation process, verifying the stepwise degradation of the target nucleosides by *H. coagulans* NJ01 (Figure S1A–D). Consistent with the retention times of hypoxanthine and guanine standards, the characteristic peaks corresponding to the biodegradation products of inosine and guanosine were detected at retention times of 4.11 min and 3.49 min, respectively (Figure S1E,F). Moreover, the absorption spectra of the inosine and guanosine biodegradation products matched those of the hypoxanthine and guanine standards, confirming their identities as hypoxanthine and guanine (Figure S1G,H). Notably, the concentrations of hypoxanthine and guanine did not exhibit a continuous decrease over time, suggesting that they are the terminal products of the pathway.

### 3.3. Characteristics of the H. coagulans NJ01 Genome

**Figure 3:**
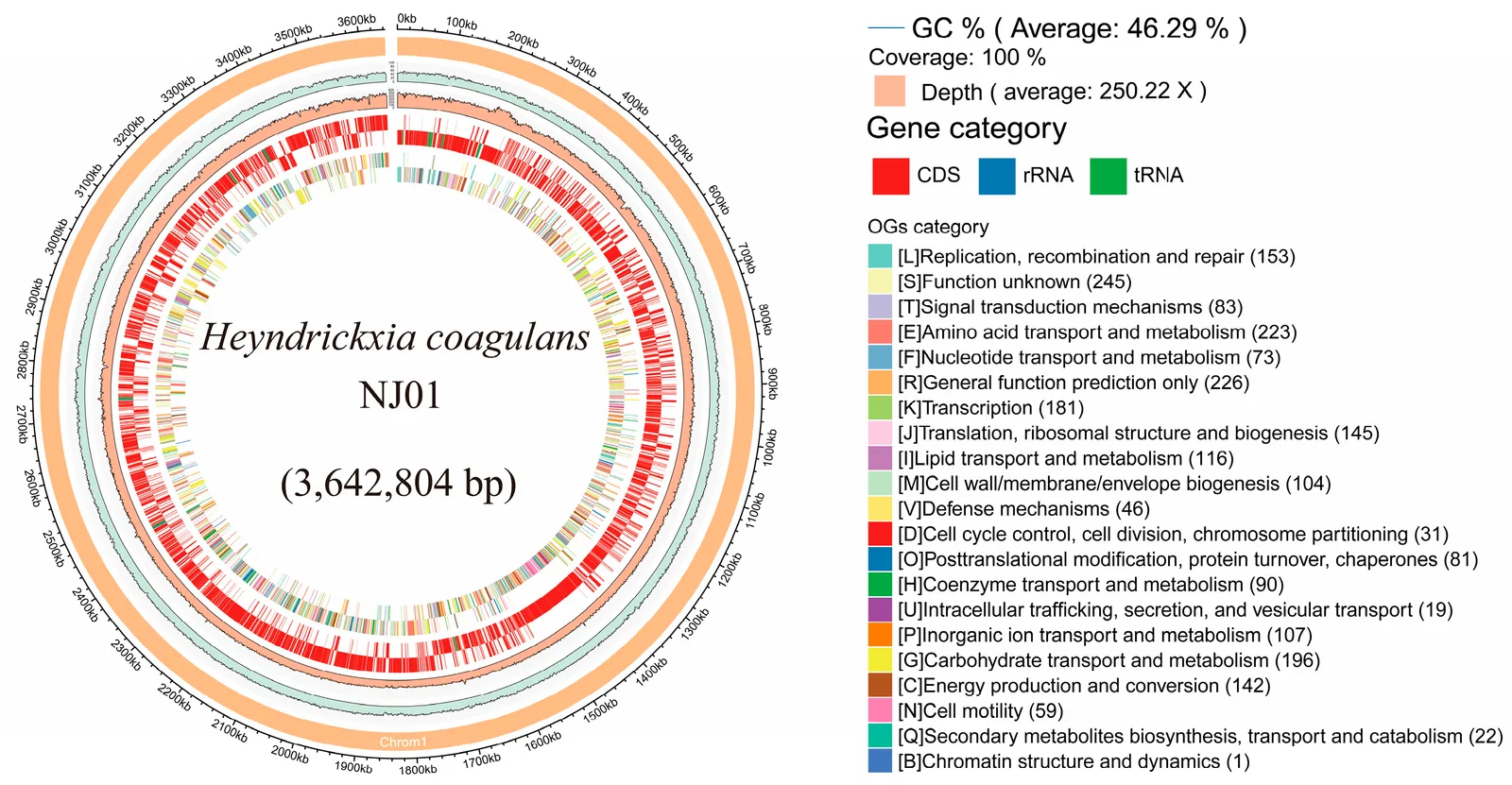
Circular genome map of *H. coagulans* NJ01.

Genome-wide analysis and annotation were performed on *H. coagulans* NJ01. Ultimately, a single contig was obtained with a total length of 3.64 Mb, and an overall GC content of 46.29%. The integrity of the genome assembly was well corroborated (sequencing depth: 250.22×; BUSCO: 100%; completeness: 99.45%; contamination: 0%). Genome annotation predicted 3475 protein-coding genes, 83 transfer RNAs (tRNAs) and 30 ribosomal RNAs (rRNAs). The Circos plot of the *H. coagulans* NJ01 genome is illustrated in Figure 3.

**Figure 4:**
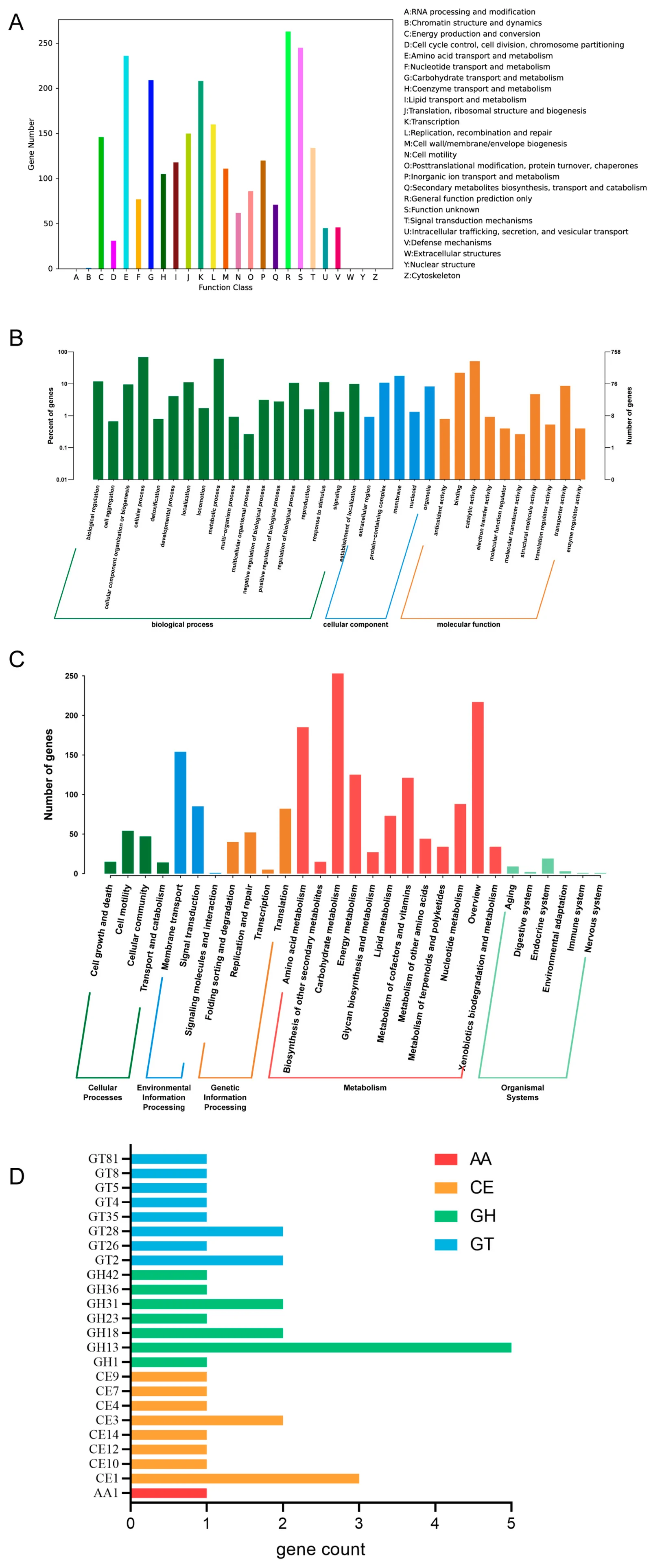
Characteristics of *H. coagulans* NJ01 genome. (**A**) Functional category distribution from COG annotation. (**B**) GO functional classification of annotated genes. (**C**) Enrichment histogram of KEGG pathways. (**D**) CAZy database annotation.

Functional classification of the protein-coding genes of *H. coagulans* NJ01 was performed using the COG database, revealing 25 distinct functional categories (Figure 4A). GO annotation covered three primary ontologies (Figure 4B): Biological Process (BP), Cellular Component (CC), and Molecular Function (MF), with 758 genes annotated in total. KEGG annotation identified 1059 genes, whose functions are predominantly enriched in metabolic pathways (Figure 4C). CAZy database annotation (Figure 4D) indicated that *H. coagulans* NJ01 contains glycosyl transferases (GT, 10 genes), glycoside hydrolase (GH, 13 genes), auxiliary activity (AA, 1 gene) and carbohydrate esterases (CE, 11 genes). Additionally, virulence factor analysis of *H. coagulans* NJ01 revealed only two genes (*clpC* and *clpP*) with a confidence level > 75%. Analysis of antibiotic resistance genes in *H. coagulans* NJ01 showed that it harbors only one antibiotic resistance gene (*ugd*) with a confidence level > 70%.

**Figure 5:**
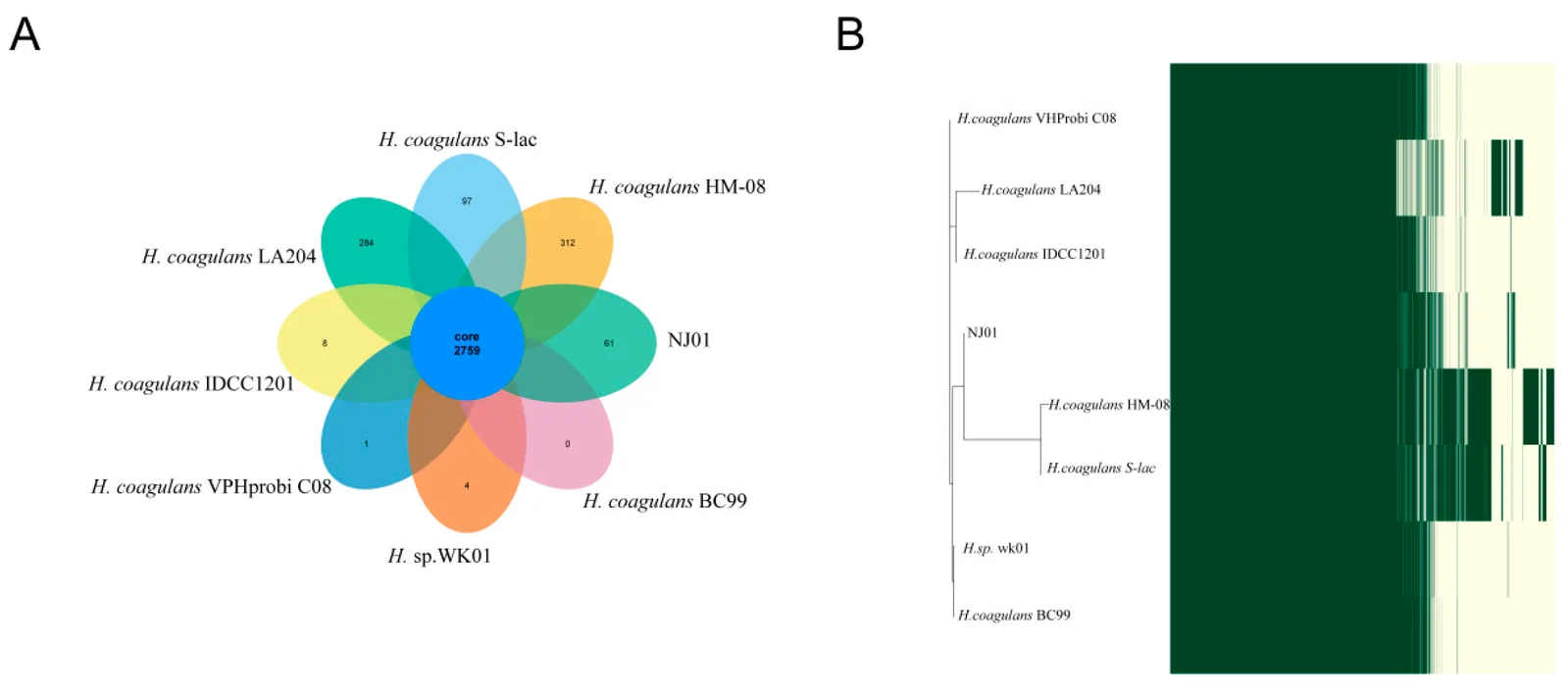
Comparative genomic and pan-genomic analysis. (**A**) Comparative analysis of genes annotated among eight *H. coagulans* strains showing different number of unique and sharing genes among the *H. coagulans* genome. (**B**) Pan-genome presented a core and accessory gene by comparing with the genomes of *H. coagulans*.

Simultaneously, a comparative genomic and pan-genome analysis was conducted to assess the diversity of genes within *H. coagulans* strains. This involved a comparison with the genomes of eight *H. coagulans* strains (Table S2). All eight *H. coagulans* strains shared 2759 genes. The numbers of genes found only in each strain from *H. coagulans* S-lac, *H. coagulans* HM-08, *H. coagulans* BC99, *H. coagulans* VHProbi C08, *H. coagulans* IDCC1201, *H. coagulans* LA204, *H.* sp. WK01, *H. coagulans* NJ01 are 97, 312, 0, 1, 8, 284, 4, and 61 (Figure 5A), respectively. Pan-genome analysis of the *H. coagulans* revealed the presence of 2759 core genes, along with 1162 shell genes and 767 cloud genes (Figure 5B).

### 3.4. Inosine and Guanosine Metabolic Pathways

**Figure 6:**
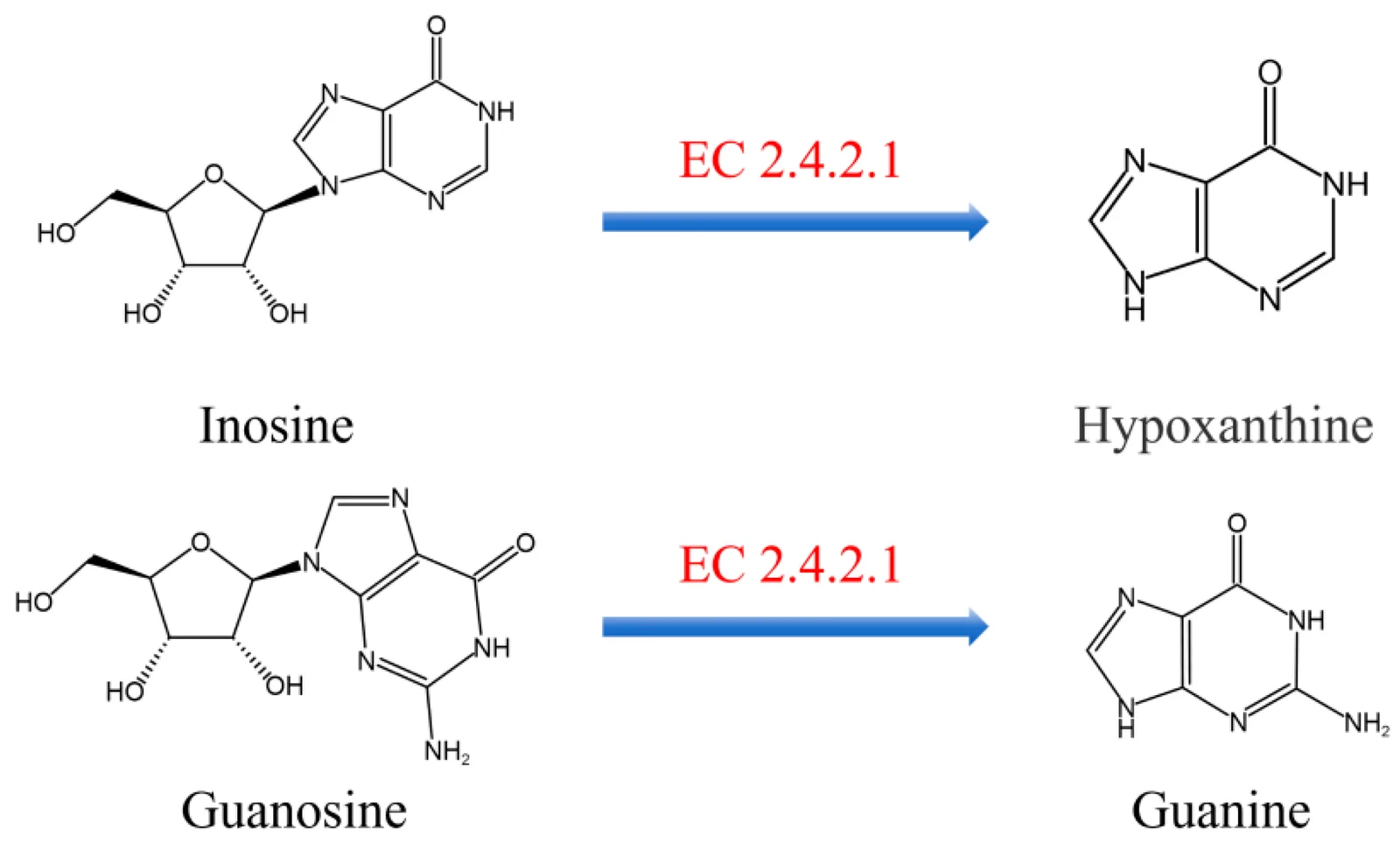
Metabolic pathway for inosine and guanosine degradation in *H. coagulans* NJ01. EC 2.4.2.1: purine-nucleoside phosphorylase.

To genetically characterize *H. coagulans* NJ01’s capacity for inosine and guanosine metabolism, we integrated whole-genome analysis with targeted identification of enzymatic genes. Purine-nucleoside phosphorylase (PNP, EC 2.4.2.1), encoded by the *deoD* gene and implicated in the catabolic pathway of inosine and guanosine, was identified via genome annotation. The key biodegradation gene in strain NJ01 was further validated by PCR. The proposed metabolic pathways and PCR validation of these key genes (Figure S1I) confirmed this strain’s genetic potential for inosine and guanosine metabolism. Based on these results, both inosine and guanosine are biodegraded through a shared degradation pathway, yielding hypoxanthine and guanine, respectively (Figure 6). Notably, the functional role of *deoD* in this process is predicted based on genomic annotation, and direct experimental validation of its gene expression analysis remains to be performed.

### 3.5. Probiotic Characteristics

**Figure 7:**
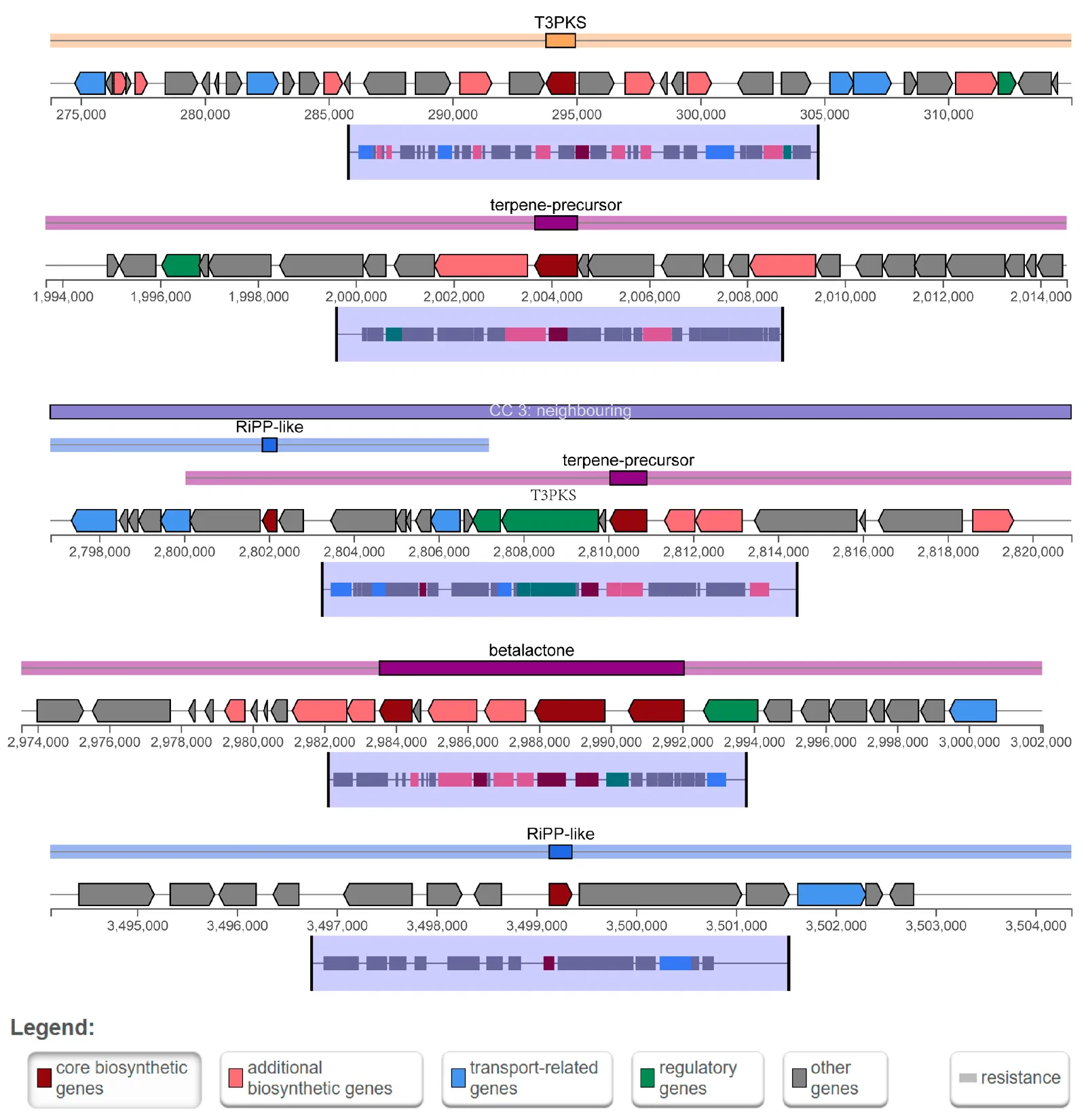
Secondary metabolite biosynthesis gene clusters in *H. coagulans* NJ01.

Based on the whole-genome sequencing data of *H. coagulans* NJ01, the genes associated with probiotic potential were systematically analyzed (Table S3) [18]. Secondary metabolite biosynthetic gene clusters (smBGCs) in *H. coagulans* NJ01 were predicted using the antiSMASH 6.0 software (Figure 7), resulting in the identification of five putative gene clusters in total (T3PKS; terpene-precursor; RiPP-like, terpene-precursor; betalactone; RiPP-like).

To investigate the gut colonization-associated surface properties of *H. coagulans* NJ01, the experimental results demonstrated that the strain exhibited affinity for ethyl acetate, with a binding capacity of 19.69%. As shown in Figure S2A, the auto-aggregation rate of *H. coagulans* NJ01 exhibited an increasing trend with prolonged incubation time, reaching 57.22% at 2 h and 63.67% at 4 h, demonstrating excellent auto-aggregation stability. In the co-aggregation assay, *H. coagulans* NJ01 formed varying degrees of co-aggregates with *S. aureus* and *E. coli*.

### 3.6. Safety Experiment Analysis

After 48 h of streak plating cultivation, the hemolytic ring results revealed that *H. coagulans* NJ01 exhibited γ-hemolysis on blood agar plates, indicating that this strain does not produce hemolysins. In contrast, *S. aureus* showed a β-hemolytic ring, forming a clear zone of hemolysis (Figure S2B). After incubation for 24 h, the color of *H. coagulans* NJ01 was not changed compared with the control group, indicating that *H. coagulans* NJ01 had no ability to produce corresponding biogenic amines (BAs) (Figure S2C). The cytotoxicity test results are shown in Figure S2D, with the survival rate of the blank control cells being 99.16% and that of the fermentation supernatant group being 99.24%. The detection results showed that distinct inhibition zones were observed around the antibiotic-impregnated disks, and the diameters of these inhibition zones all fell within the susceptible range specified by CLSI. Specifically, *H. coagulans* NJ01 exhibited a susceptible phenotype to the tested antibiotics (Table S4).

## 4. Discussion

*H. coagulans* (formerly *Bacillus coagulans* and *Weizmannia coagulans*) is a spore-forming, thermotolerant, and acid-resistant bacterium that combines the probiotic properties of lactobacilli with the resilience of bacilli. Recognized as Generally Recognized as Safe (GRAS) by the U.S. Food and Drug Administration (FDA) and approved for use in food in China, *H. coagulans* has found widespread application in functional foods, animal feed, and dietary supplements due to its stability and health-promoting attributes [28]. In the food industry, specific examples include *H. coagulans* MTCC 5856 [29], which is incorporated into baked goods such as waffles and fermented dairy products, and *H. coagulans* BC4 [30], which is utilized in functional food formulations like dried jujube paste. In the animal husbandry sector, *H. coagulans* TBC169 [31] has been demonstrated to improve the body weight and intestinal morphology of broiler chickens. Furthermore, strains including *H. coagulans* BC01 [32] and *H. coagulans* BC30 [33] play pivotal roles in promoting intestinal health and boosting host immune competence. Recent studies suggest that specific strains of *H. coagulans* including *H. coagulans* BC99 [10] and *H. coagulans* TBC169 [11], may ameliorate HUA by modulating purine metabolism and enhancing intestinal uric acid excretion. Here, we preliminarily find that a novel strain of *H. coagulans* NJ01 capable of efficiently biodegrading inosine and guanosine in vitro was successfully isolated from *Jiaosu* for the first time. Compared with BC99 and TBC169, NJ01 was isolated from *Jiaosu* rather than animal-derived samples. In addition to its capacity for inosine and guanosine biodegradation, genomic discrepancies also exist among these strains, suggesting strain-specific genetic backgrounds.

Inosine and guanosine, as precursors of uric acid, are important contributing factors to HUA and gout [17]. Intestinal epithelial cells exhibit significantly lower absorption capacity for purines compared to nucleosides [34]. Accumulating evidence has preliminarily confirmed that probiotics capable of biodegrading nucleosides exert beneficial effects in alleviating HUA [35]. Regarding nucleoside biodegradation, PNP is the principal enzyme. Strains such as *Pediococcus acidilactici* GR-5 [9] convert inosine and guanosine to hypoxanthine and guanine, respectively. Our in vitro assays showed that *H. coagulans* NJ01 efficiently biodegraded inosine and guanosine within 24 h using resting cells and CEs, suggesting its potential as a candidate for HUA intervention. HPLC and UV spectral analyses identified the conversion of inosine to hypoxanthine and guanosine to guanine. Furthermore, the key biodegradation gene in *H. coagulans* NJ01 was further confirmed by PCR.

The evaluation of stress tolerance and secondary metabolite profiles is conducive to exploring its probiotic potential. Five secondary metabolite biosynthetic gene clusters were identified in the genome of *H. coagulans* NJ01. The T3PKS cluster (273,778–314,962 nt, 41,185 nt) putatively catalyzes iterative decarboxylative condensation of malonyl-CoA with starter CoA thioesters to generate diverse polyketide scaffolds. Other detected smBGCs included a terpene-precursor cluster (1,993,664–2,014,533 nt, 20,870 nt), a RiPP-like terpene-precursor cluster (2,796,849–2,820,912 nt, 24,064 nt), a betalactone cluster (2,973,547–3,002,047 nt, 28,501 nt), and a RiPP-like cluster (3,494,132–3,504,359 nt, 10,228 nt). All smBGCs represent in silico predictions without experimental metabolite validation [36,37,38,39]. Gut colonization ability is a prerequisite for probiotics to exert their probiotic effects [40,41]. Based on our results of auto-aggregation, co-aggregation and cell-surface hydrophobicity, strain *H. coagulans* NJ01 exhibits strong auto-aggregation capacity, which may contribute to its intestinal colonization potential.

Hemolytic activity is a critical indicator for evaluating the potential pathogenicity of microorganisms [42]. Experimental results demonstrated that strain *H. coagulans* NJ01 exhibited no hemolytic activity, which preliminarily confirms its lack of red blood cell-damaging capacity and indicates an absence of significant invasive potential and pathogenic risk. BAs are naturally occurring alkaline nitrogen-containing thermostable organic compounds, which are formed by the decarboxylation of amino acids [43]. Appropriate concentrations of BAs exert beneficial effects on the human body, whereas excessive levels can induce adverse reactions such as allergies and vomiting [44]. In decarboxylase assays, *H. coagulans* NJ01 showed negative reactions to arginine, lysine, and ornithine, indicating that this strain lacks the capacity to produce the corresponding biogenic amines. Caco-2 cell cytotoxicity assays can preliminarily validate that probiotics have no direct toxic effects on intestinal epithelial cells [26]. Antibiotic susceptibility is a critical indicator for evaluating the safety of probiotics. Comprehensive in vitro assays demonstrated that *H. coagulans* NJ01 exhibited negligible antibiotic resistance, thereby complying with the fundamental safety criteria for ideal probiotic strains [24]. Notably, genes including *clpC*, *clpF*, and *ugd* are involved in fundamental cellular processes rather than bona fide virulence factors. The inferred low-virulence risk of *H. coagulans* NJ01 is mainly based on the genome-wide absence of known virulence-determinant genes. Since Firmicutes are intrinsically insensitive to polymyxin, the contribution of *ugd* to polymyxin resistance is minor [24,25].

## 5. Conclusions

In this study, a novel strain of *H. coagulans* NJ01 capable of efficiently biodegrading inosine and guanosine was successfully isolated from *Jiaosu*. Comprehensive genotype and phenotype evaluations preliminarily confirmed its probiotic potential and safety, supporting its candidacy for further development. Subsequent studies will focus on gastrointestinal tolerance assessment, establishment of HUA-affected mouse models to evaluate the amelioration of HUA and related physiobiochemical parameters. Combined with further mechanistic exploration, these investigations will provide critical evidence to advance NJ01 toward functional-food or live-biotherapeutic applications for hyperuricemia intervention.

## Data Availability

The original contributions presented in this study are included in the article/Supplementary Materials. Further inquiries can be directed to the corresponding author.

## Supplementary Materials

The following supporting information can be downloaded at: https://www.mdpi.com/article/10.3390/foods15183254/s1, Table S1: Primers for target gene PCR; Figure S1: HPLC chromatogram analysis of nucleoside (inosine and guanosine) biodegradation by *H. coagulans* NJ01 and PCR validation of the key biodegradation gene in *H. coagulans* NJ01. (A), (C) Nucleoside biodegradation and metabolite production at 0 h. (B), (D) Nucleoside biodegradation and metabolite production at 24 h. (E) Hypoxanthine standard. (F) Guanine standard. (G) UV scanning spectra comparison of the inosine biodegradation products by *H. coagulans* NJ01 versus the hypoxanthine standard. (H) UV scanning spectra comparison of the guanosine biodegradation products by *H. coagulans* NJ01 versus the guanine standard. (I) corresponding functional gene expression of *H. coagulans* NJ01. Lanes 1 and 2: NJ01-03200 (*deoD*, 702 bp); Table S2: Reference strains used for Comparative and average nucleotide identity (ANI) analyses; Table S3: Probiotic Properties and Safety-Related Genes in *H. coagulans* NJ01; Figure S2: Evaluation of probiotic characteristics and safety. (A) aggregation capacity analysis. (B) Hemolytic Test. (C) Biochemical Identification (D) Results of Caco-2 cell cytotoxicity experiments; Table S4: Evaluation of drug resistance of *H. coagulans* NJ01.
