# Supplementary material for: Pathway and Mechanism for the Biodegradation of Inosine and Guanosine by Heyndrickxia coagulans NJ01
Source: Foods. 2026 Sep 15;15(18):3254. doi: 10.3390/foods15183254 (PMC13605953; doi:10.3390/foods15183254)
Supplement: Supplementary file 1 [file foods-15-03254-s001.zip › foods-4537926-supplementary.pdf]

Table S1. Primers for target gene PCR

Gene ID	Gene name	Direction (5′→3′)	Sequence
Chrom1_03200	*deoD*	Forward primer	CACAGCAGCGGCCTGGTGCCGCGCGGCAG<br>CCATATGATGAGCACACATATTGGCGCAAA
		Reverse primer	CCGGATCTCAGTGGTGGTGGTGGTGGTGCT<br>CGAGTTACTTTACAGCAGCATCTAAAGCA

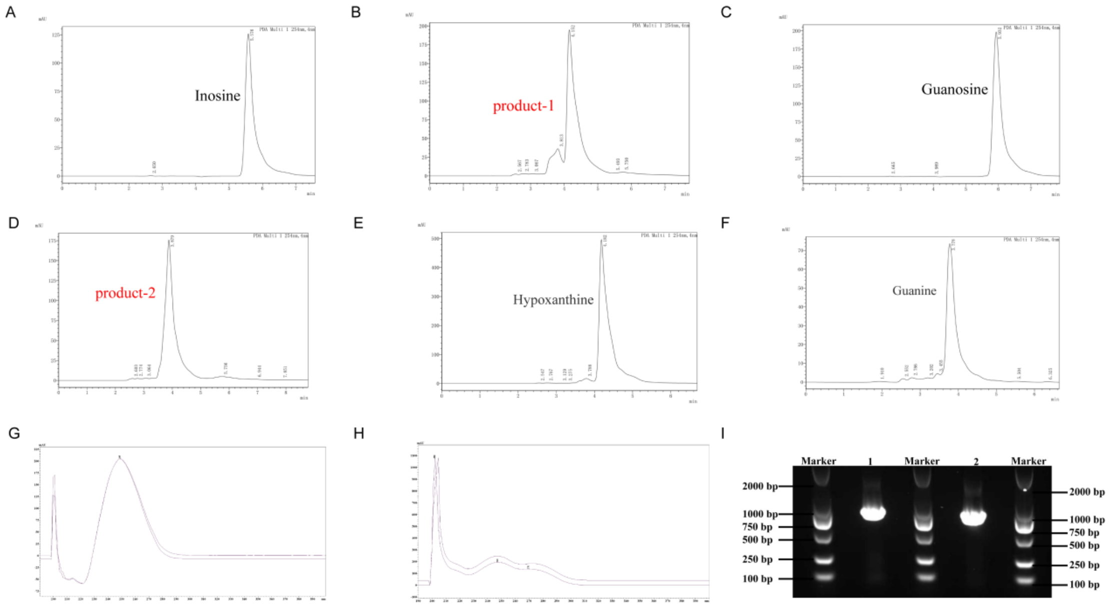

Figure S1. HPLC chromatogram analysis of nucleoside (inosine and guanosine) biodegradation by *H. coagulans* NJ01 and PCR validation of the key biodegradation gene in *H. coagulans* NJ01. (A), (C) Nucleoside biodegradation and metabolite production at 0 h. (B), (D) Nucleoside biodegradation and metabolite production at 24 h. (E) Hypoxanthine standard. (F) Guanine standard. (G) UV scanning spectra comparison of the inosine biodegradation products by *H. coagulans* NJ01 versus the hypoxanthine standard. (H) UV scanning spectra comparison of the guanosine biodegradation products by *H. coagulans* NJ01 versus the guanine standard. (I) corresponding functional gene expression of *H. coagulans* NJ01. Lanes 1 and 2: NJ01-03200 (*deoD*, 702 bp).

Table S2. Reference strains used for Comparative and average nucleotide identity (ANI) analyses.

NCBI strain identifiers	Accession	Genome size (Mb)	CDS	Used for ANI	Used for Comparative
Heyndrickxia coagulans HM-08	GCA_000876545.1	3.6	4233	No	Yes
Heyndrickxia coagulans S-lac	GCA_001039495.1	3.7	4088	Yes	Yes
Heyndrickxia coagulans LA204	GCA_003184245.1	3.6	3321	No	Yes
Heyndrickxia coagulans IDCC1201	GCA_004114715.1	3.7	3237	No	Yes
Heyndrickxia coagulans VHProbi C08	GCA_019443305.1	3.7	3238	Yes	Yes
Heyndrickxia sp. WK01	GCA_025905465.1	3.7	3308	No	Yes
Heyndrickxia coagulans BC99	GCA_029952375.1	3.7	3273	Yes	Yes
Heyndrickxia coagulans 36D1	GCF_000169195.2	3.6	3290	Yes	No
Heyndrickxia coagulans BC01	GCA_015655285.1	3.6	3146	Yes	No
Heyndrickxia coagulans DSM 1 = ATCC 7050	GCA_000832905.1	3.4	3253	Yes	No
Heyndrickxia oleronia EISK28	GCA_052044255.1	5.1	4893	Yes	No
Bacillus subtilis 168	GCA_000009045.1	4.2	4243	Yes	No

Table S3. Probiotic Properties and Safety-Related Genes in *H.coagulans* NJ01

Tolerance Items	Gene ID	Start	End	Length (bp)	Gene s	Product
Acid	Chrom1_03471	3435026	3436540	1515	*atpA*	ATP synthase subunit alpha
Acid	Chrom1_03475	3438080	3438796	717	*atpB*	ATP synthase subunit a
Acid	Chrom1_03468	3432092	3432502	411	*atpC*	ATP synthase epsilon chain
Acid	Chrom1_03469	3432525	3433946	1422	*atpD*	ATP synthase subunit beta
Acid	Chrom1_03474	3437802	3438020	219	*atpE*	ATP synthase subunit c
Acid	Chrom1_03473	3437097	3437618	522	*atpF*	ATP synthase subunit b
Acid	Chrom1_03470	3434022	3434879	858	*atpG*	ATP synthase gamma chain
Acid	Chrom1_03472	3436564	3437100	537	*atpH*	ATP synthase subunit delta
Acid	Chrom1_01481	1510623	1511690	1068	*recA*	Protein RecA
Acid	Chrom1_02052	2034875	2035996	1122	*gcvT*	Aminomethyltransferase
Acid	Chrom1_02618	2570004	2570387	384	*gcvH*	Glycine cleavage system H protein
Acid	Chrom1_02821	2768194	2772756	4563	*gltB*	Glutamate synthase [NADPH] large chain
Acid	Chrom1_02355	2318635	2320395	1761	*pyk*	Pyruvate kinase
Acid	Chrom1_02457	2428079	2429290	1212	*metK*	S-adenosylmethionine synthase
Bile	Chrom1_01793	1812347	1812931	585	*plsC*	1-acyl-sn-glycerol-3-phosphate acyltransferase
Bile	Chrom1_00139	124430	124855	426	*rplK*	50S ribosomal protein L11
Bile	Chrom1_00143	126442	126807	366	*rplL*	50S ribosomal protein L7/L12

Bile	Chrom1_00154	141212	141835	624	*rplD*	50S ribosomal protein L4
Bile	Chrom1_00170	148233	148733	501	*rplE*	30S ribosomal protein S5
Bile	Chrom1_00168	147279	147815	537	*rplF*	50S ribosomal protein L6
Bile	Chrom1_01387	1414886	1415230	345	*rplS*	50S ribosomal protein L19
Bile	Chrom1_02335	2297138	2297491	354	*rplT*	50S ribosomal protein L20
Bile	Chrom1_02226	2188581	2188889	309	*rplU*	50S ribosomal protein L21
Bile	Chrom1_00584	564636	565340	705	*nagB*	Glucosamine-6-phosphate deaminase 1
Bile	Chrom1_01087	1116803	1117735	933	*oppB*	Oligopeptide transport system permease protein OppB
Bile	Chrom1_01088	1117732	1118751	1020	*oppC*	Oligopeptide transport system permease protein OppC
Bile	Chrom1_01089	1118770	1119831	1062	*oppD*	Oligopeptide transport ATP-binding protein OppD
Bile	Chrom1_01090	1119833	1120774	942	*oppF*	Oligopeptide transport ATP-binding protein OppF
Bile	Chrom1_01230	1259012	1260853	1413	*pdhD*	Dihydrolipoyl dehydrogenase
Bile	Chrom1_02224	2187936	2188226	291	*rpmA*	50S ribosomal protein L27
Bile	Chrom1_01366	1394430	1394618	189	*rpmB*	50S ribosomal protein L28
Bile	Chrom1_02336	2297525	2297722	198	*rpmI*	50S ribosomal protein L35
Bile	Chrom1_01929	1924787	1925923	1137	*rpsA*	30S ribosomal protein S1
Bile	Chrom1_01436	1459217	1459972	756	*rpsB*	30S ribosomal protein S2
Bile	Chrom1_00159	143687	144343	657	*rpsC*	30S ribosomal protein S3
Bile	Chrom1_01455	1481195	1481464	270	*rpsO*	30S ribosomal protein S15

Bile	Chrom1_01382	1412423	1412695	273	*rpsP*	30S ribosomal protein S16
Bile	Chrom1_02123	2098623	2098883	261	*rpsT*	30S ribosomal protein S20
Bile	Chrom1_02109	2082917	2083102	186	*rpsU*	30S ribosomal protein S21
Bile	Chrom1_02721	2679446	2680375	930	*ppaC*	Manganese-dependent inorganic pyrophosphatase
Bile	Chrom1_01854	1856890	1858227	1338	*glnA*	Glutamine synthetase
Bile	Chrom1_02963	2913944	2914414	471	*luxS*	S-ribosylhomocysteine lyase
Bile	Chrom1_03501	3461645	3463246	1602	*pyrG*	CTP synthase
Bile	Chrom1_03512	3480390	3482060	1671	*argS*	Arginine--tRNA ligase
Gut persistence	Chrom1_00633	619194	619526	333	*celA*	PTS system cellobiose-specific EIIB component
Gut persistence	Chrom1_00634	619544	619873	330	*celD*	PTS system cellobiose-specific EIIA component
Temperature	Chrom1_00188	160151	161263	1113	*katE*	Catalase HPII
Oxidative	Chrom1_01595	1627652	1628590	939	*trxB*	Thioredoxin reductase
Oxidative	Chrom1_03100	3061784	3062257	474	*gpx*	Hydroperoxy fatty acid reductase gpx1
Lactate	Chrom1_02116	2089084	2090910	1827	*dnaK*	Chaperone protein DnaK
Lactate	Chrom1_02350	2312866	2313801	936	*mdh*	Malate dehydrogenase

Note: Genes listed in this table are predictive and not phenotypically validated in the present study.

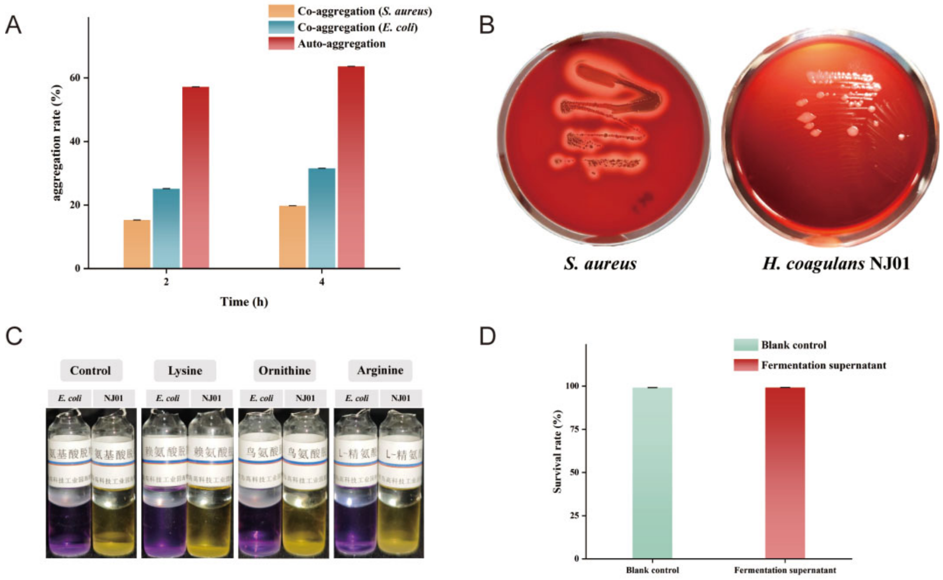

Figure S2. Evaluation of probiotic characteristics and safety. (A) aggregation capacity analysis. (B) Hemolytic Test. (C) Biochemical Identification (D) Results of Caco-2 cell cytotoxicity experiments.

Table S4. Evaluation of drug resistance of *H.coagulans* NJ01.

Antibiotic	Standard (μg/slice)	Diameter (mm)	type
C	30	48±1.5	S
S	10	40±1.15	S
CC	2	50±1.25	S
AMP	10	40±1.15	S
GEN	10	36±1.25	S
TET	30	48±0.75	S
KAN	30	40±1.0	S
ROX	15	42±1.2	S
CIP	5	40±1.25	S
